# An in-depth analysis of nucleation and growth mechanism of CdS thin film synthesized by chemical bath deposition (CBD) technique

**DOI:** 10.1038/s41598-022-19340-z

**Published:** 2022-09-12

**Authors:** A. S. Najm, Hasanain Salah Naeem, Hasan Sh. Majdi, Siti Aishah Hasbullah, Hiba Ali Hasan, K. Sopian, Badariah Bais, Heidar J. Al-Iessa, Hayder A. Dhahad, Jamal M. Ali, Abbas J. Sultan

**Affiliations:** 1 Department of Electrical, Electronic and System Engineering, Faculty of Engineering and Built Environment, Universiti Kebangsaan Malaysia (UKM), 43600 Bangi, Selangor Malaysia; 2 University of Al Muthanna, Samawah, Iraq; 3 Department of Chemical Engineering and Petroleum Industries, Al-Mustaqbal University College, Babylon, 51001 Iraq; 4 Department of Chemical Sciences, Faculty of Science and Technology, Universiti Kebangsaan Malaysia (UKM), 43600 UKM Bangi, Selangor Malaysia; 5 Department of Pharmacognosy and Medicinal Plants, College of Pharmacy, Mustansiriyah University, Baghdad, Iraq; 6 Solar Energy Research Institute (SERI), Universiti Kebangsaan Malaysia (UKM), 43600 Bangi, Selangor Malaysia; 7 Oil Exploration Laboratories, Al Wazireya, Baghdad, Iraq; 8 Mechanical Engineering Department, University of Technology, Baghdad, Iraq; 9 Chemical Engineering Department, University of Technology, Baghdad, Iraq

**Keywords:** Materials science, Nanoscience and technology

## Abstract

The aim of this study is to acquire a deeper understanding of the response mechanism that is associated with the formation of CdS thin films. We presented an effective and new hybrid sensitisation technique, which involved the 1-step linker between the related chemical bath deposition (CBD) process and the traditional doping method during CBD for synthesising high-quality, CdS thin films. The mechanism for the combined synthesis of the films is also describes. CdS films were electrostatically bonded to soda-lime glass, causing the formation of the intermediate complexes [Cd(NH_3_)_4_]^2+^, which aided in the collision of these complexes with a soda-lime glass slide. In the one-step fabrication technique, 3-Mercaptopropionic Acid (MPA) was employed as a second source of sulphur ions and a linker molecule. Optical studies showed that the bandgap ranged between (2.26–2.52) eV. CdS + MPA films exhibited a uniform distribution of spherical molecules based on their morphological properties. After annealing, this approach significantly altered the electrical characteristics of CdS films. The CdS + MPA films displayed the highest carrier concentration whereas the CdS + Ag + MPA films exhibited the lowest resistivity, with a jump of 3 orders of magnitude.

## Introduction

It is well-known that transferring a chemical process from its analytical modelling stage to actual synthesis is rather difficult. This can be attributed to the fact that the metal nanoparticles can spontaneously coagulate and aggregate together owing to their highly dispersed state, and hence, have to be stabilised^1,2^. Many researchers have determined the effect of using bi-functional bridging ligands on the quantum dots for developing a defined and more rigid system. In this study, we have proposed a new technique, wherein we used a bi-functional linker molecule for selectively attaching the pre-synthesised metal chalcogenide QDs (e.g., CdS, CdSe) to the oxide surfaces (e.g., TiO_2_ or ZnO). Thus, this technique allowed better sub-monolayer coverage with a minimum particle aggregation. This helped in accurately controlling the size and spectral absorption properties of these QDs^3^. Some earlier reports showed that the semiconductor molecules such as CdS was a prospective buffer layer that could be employed as typical n-type heterojunction partners in current and upcoming thin-film PV devices due to its direct bandgap transition (Eg 2.4 eV), transparency, n-type conductivity, and a direct bandgap transition high electron affinity (4.2 eV)^4^. In order to improve the CdS thin film characterization, we took advantage of the QDs and attempted to stabilise the CdS nanocrystal thin film surfaces via the use of appropriate organic compounds known as capping agents. These might be used throughout the synthesis process and attach to the particle surfaces; as a result, the growth of the particles would be decelerated, and aggregation would be avoided. In addition to the prevention of the nanoparticle aggregation, any modification to the metal surfaces by the organic ligands can affect the QD properties such as electrical conductivity and the PhotoLuminescence (PL). Since the dynamics of photogenerated excitons are impacted by the electronic states in the surrounding environment, such as ligands, this may be explained by the higher carrier transfer rate between the QDs as a result of the ligand exchange^5^. In a related report, Kovalenko et al. (2009) noticed that when they employed molecular metal chalcogenide surface ligands nearby to the QDs, they were capable of maintaining the size-dependent optical absorption characteristics of the molecules while considerably improving the electron mobility^6^.

Ligands also play a vital role and affect the shape, size, growth mechanism, crystal structure and electron transfer reactions^7–10^. In their study, Yu et. al (2012)^11^ suggested the in-situ Linker-Assisted Chemical Bath Deposition (LACBD) method for achieving photostable CdSe/CdS QD-sensitized TiO_2_ surfaces utilising a bi-functional modifier, i.e., ThioGlycolic Acid (TGA). Due to the stabilising nature of the TGA, the QDs synthesised using this method were smaller in size and had a narrower size distribution than those synthesized using the conventional CBD method. Until recently, thiols were regarded as the most effective ligands for directing the growth and nucleation of II-VI semiconductor nanocrystals^12^. Among the many thiol-based ligands, those containing a mercapto-group and one carboxyl group attached to an alkyl chain have been used most commonly. It is observed that 3-Mercaptopropionic Acid (MPA) is an organic molecule having two functional groups. The coordination between one or both of these functional groups and the nanoparticle surfaces has two advantages: (1) Passivation of the dangling bonds to the nanoparticle surface; and (2) Protection of nanoparticles and inhibition of their attraction to one another, which prevents aggregation.

MPA is a preferred ligand because its use resulted in a low-density mid-gap state, which enables the capture of charge carriers across long distances beyond their depletion region^13^. Regarding the usage of CdS films as a buffer layer, it has been noted that these films must be very thin in in order to maintain low series resistance and high photon transmission. This permitted the optimization of minority carrier transport. Nonetheless, thicker CdS films induced a Schottky barrier effect and enhanced minority carrier transmission^14^. Doping semiconductors after the incorporation of acceptors or donors into the crystal lattice was a conventional strategy for reducing electrical resistivity^15^. This doping may be accomplished utilising an in-situ chemical method during crystal formation, in which a particular volume of a salt solution containing doping atoms was introduced to the reaction solution without affecting the crystal lattice structure. Numerous researchers have tried to examine and design a modified CdS bandgap over the last few years by identifying point defects and doping techniques that increase the absorption of incident light. Popular doping elements for CdS films included indium, tin, copper, gallium, aluminium, and magnesium^15–20^.

Silver (Ag) was a Group I element that enhanced the electrical characteristics of (II-VI) semiconductors by acting as a donor dopant. This doping atom could be introduced into CdS nanoparticles without changing the crystal lattice structure of the nanoparticles. The observed potential difference between the conduction band of CdS and the Fermi level of Ag promoted electron transfer between the doped material and the semiconductor matrix^21^. Sergio et al. (2014) observed that the roughness and bandgap energy increased with increasing Ag concentration, up to Cd depletion. The subsequent formation of silver sulphide (Ag_2_S) resulted in a reduction in roughness and bandgap energy with increasing AgNO_3_ concentration^22^. Pacheco et al. (2017) also observed that the polycrystalline structure of the Ag^+^ doped material could significantly affect the quantum confinement and lead to a decrease in their mean particle size to 4.12 nm from 5.46 nm, which increased the energy emission because of the decreasing particle sizes below the CdS exciton Bohr radius^23^.

Ristova and Ristov (1998), examine the influence of Ag as a dopant in CdS-thin films on the cell parameters of CdS based PV cells, and they found an enhancment in efficiency aroung 2.66 compare without doping^24^. While Saikia et al. (2011) demonstrated that the addition of silver to CdS/PVA nanocomposite thin film enhanced the cell's conversion efficiency from 4.92 to 5.63%^25^. In addition, Mehmood et al. (2021) fabricated CISe/Ag–CdS innovative structure devices and shown dramatically enhanced optoelectronics capabilities, with PCE reaching 3.34% in CISe/Ag–CdS co-sensitized solar cells^26^.

Though many research studied the doping by Ag atoms, it was shown that the surface of all doped films had aggregates, which was typically seen when the materials were formed using the CBD approach. In this work, we suggest a new approach for achieving high CdS thin films by combining the Linker-Assisted CBD (LACBD) technology with doping through the CBD process. We also revealed the mechanism by which the formation of thin CdS films was inhibited.

## Experimental method

### CdS thin film deposition by the LACBD process

Based on the particular product, the experiment could employ a broad range of precursors and synthesis conditions. The approach used here consists of two experimental steps. CdS was synthesised in Stage 1 using several techniques, including standard CdS thin films, MPA-sensitized CdS, and Ag-doped CdS. The second stage included the optimization of CdS by the hybrid method, which consisted of the mixing of AgNO_3_ and MPA in a single step. We basically prepared two stock solutions for each strategy. Silver Nitrate (AgNO_3_) was dissolved in deionized water (50 ml) to form a 0.01 M silver nitrate solution for the preparation of Ag-doped CdS thin films. Then, MPA stock solution was made by dissolving MPA (0.212 g, 2 mmol) in mixture of (10 ml methanol + 3 ml water). Using KOH, the pH of the solution has been adjusted to 10^27^. According to our prior work, the standard CdS thin film was synthesised^28^. In this method, we ultrasonically cleaned and degreased a 25 mm 25 mm soda lime glass slide substrate. The chemical bath was prepared with deionized water and a solution of ammonium hydroxide by volume (10:1 v/v). Sources of sulphur and cadmium salts were Thiourea (0.002 M) and Cadmium Sulphate (0.002 M), respectively. For preparing Ag-doped CdS thin films, AgNO_3_ (0.8 ml of 0.01 M stock solution) was slowly added to the CdSO_4_ solution in a growth beaker, and the mixture was constantly and vigorously stirred for 10 min. MPA films were prepared in the following manner. Initially, we conducted the CdS reaction, wherein we added (0.1 M) in a growth beaker, 20 min after the CdS synthesised. On the other hand, in Stage 2, the CdS synthesis is initiated after adding AgNO_3_ in the CdSO_4_ solution. Then, added MPA (0.1 M) to the mixture, 20 min after the initiation of the complete reaction. According to Sandoval and Ramrez (2009), who studied the early growth phases of CdS thin films during their chemical deposition, MPA was introduced after 20 min. They observed that growth occurred at deposition durations ranging from 15 to 18 min, resulting in a thick and compact CdS inner layer^29^. In order to optimise the growth mechanisms of CdS thin films, MPA was added to the chemical reaction 20 min after it started. Thereafter, the reaction was carried out as mentioned above. All the experiments were conducted at the specified deposition temperature (80 °C) for 30 min, under vigorous and constant stirring.

### The characterisation of thin film

With the aid of a Lambda 950 UV /VIS /NIR spectrometer, optical characteristics were determined in the wavelength range of 400 to 700 nm (Perkin-Elmer, USA). At room temperature, the structural characteristics of the films were evaluated using an AXS-D8 Advance Cu-K diffractometer (Bruker Corp., USA). Using the Cu-K radiation wavelength,, of 1.5408, we also examined the XRD patterns in a 2 range with a step size of 0.02°, ranging 10° to 80°. FEI Quanta 400F field emission scanning electron microscope (FESEM) equipped with Oxford- Instruments INCA 400 X-Max detector for energy–dispersive x-ray spectroscopy (EDX) measurements at × 300 magnification (1 mm × 1 mm spot size) and 20 kV accelerating voltage. Finally, the electrical characteristics of films were examined using an HMS ECOPIA 3000 Hall Effect measuring instrument with a 0.57 T magnetic field and 45 nA probe current. We used the Ag paste method to form an ohmic contact by adding Ag dots to each sample's four corners, and then repeated each reading for each sample ten times to improve the reliability of our findings.

## Results and discussion

### Optical properties

**Figure 1:**
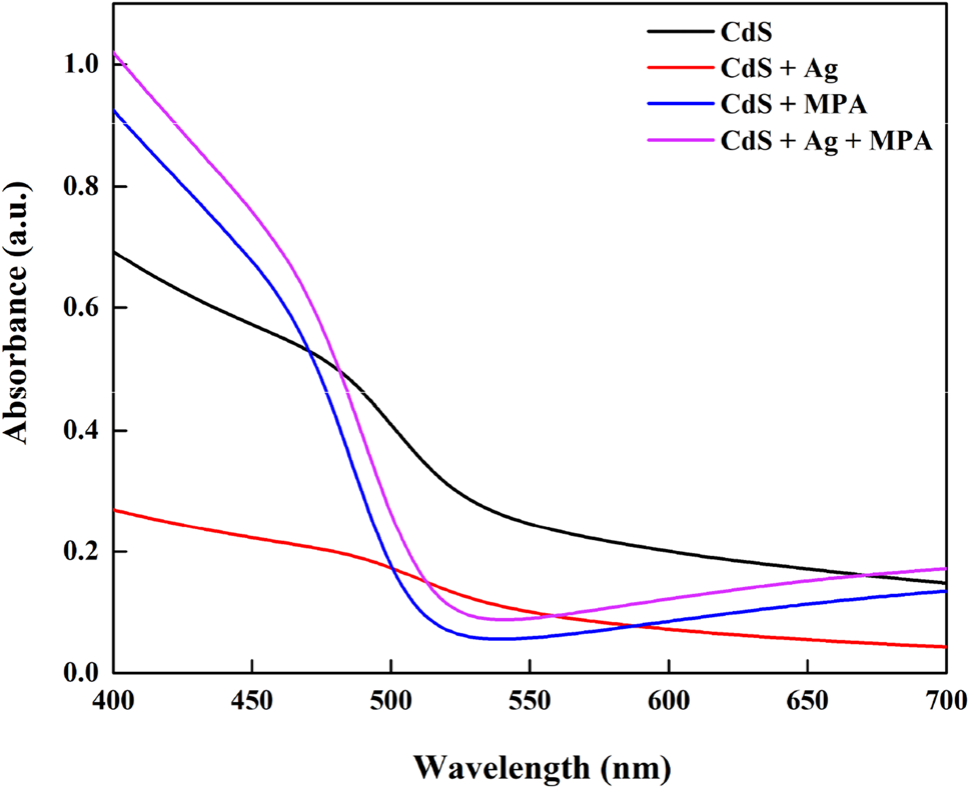
UV–Vis absorption spectra of the CdS thin films.

In an earlier study, it was stated that the absorption spectra having a distinctive peak and a sharp onset indicated a small size distribution^30^. All these features are not noted when the CdS thin films are obtained from direct growth. The relative absorption of visible light by the CdS films could reveal a significant amount of information, as shown in Fig. 1.

**Figure 2:**
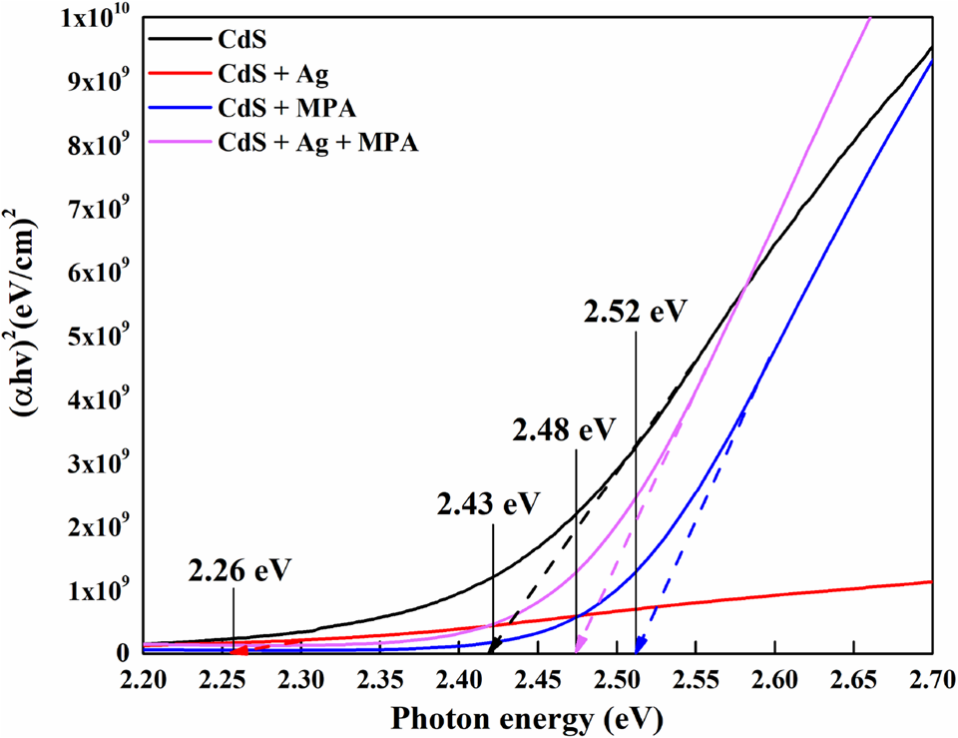
Difference of (*αhν*)^2^ with photon energy (*hν*) for CdS thin films.

As the absorbance was directly associated with the quantity of the CdS sensitizer that was uploaded, we concluded that the CdS film was based on the following order: CdS + MPA > dS + MPA + Ag > CdS > CdS + Ag, as derived from the absorption spectra. The absorption edge of pure CdS is about 510 nm, however the absorption potential in the visible light range of CdS + MPA is improved compared to pure CdS, and the absorption edge shifts to 492 nm, that may be due to the adhesion of the MPA molecules to the metal centres present on the nanoparticle surfaces, increased the electron density of the solid, which was based on the energy levels in the valence bands (Jiménez-hernández et al., 2016). CdS + Ag + MPA has also showed improvment in regard to absorption edge at about 500 nm, attributed to the decrease of the electron concentration in the valence bands (Unni, Philip and Gopchandran, 2008). Whereas, CdS + Ag displayed the lowest absorption edge at about 548 nm, probably due to the introduction of an additional energy level near the bottom of conduction band. The bandgap was estimated using the Tauc plot, which is used to calculate the optical bandgap of semiconductors. A Tauc plot typically depicts the amount hv (photon energy) on the abscissa and the quantity (αhν)^1/2^ on the ordinate, where is the material's absorption coefficient. As a result, extrapolating this linear area to the abscissa reveals the energy of the CdS optical bandgap^31^ as shown in Fig. 2.

The above results indicated a decrease in the bandgap after the Ag doping. This was attributed to the development of additional energy levels in bandgaps. This led to the narrowing of the bands and a subsequent decrease in the bandgaps^32^. A similar decrease was noted in the bandgap when Ag ions were doped onto CdS thin films, which were prepared using the Close Spaced Sublimation (CSS) technique, as described by Muneeb et al.^33^.

A small blue shift has been found in CdS + MPA sample, which we noted as a possible quantum confinement after the addition of MPA (0.1 M) due to the increasing value of Eg (2.52 eV)^34^. Comparing the bandgap value of bulk CdS (2.43 eV) to the limited particle size formed by the addition of 0.1 M MPA concentration confirms quantum confinement^34^. This quantum confinement effect is related to the modification of electronic characteristics by shifting the energy level positions of the conduction and valence bands to more negative and positive values, respectively. This change in redox potential favours electron transfer and improves photoactivity^35^. Further details related to impact of this phenomena can be noticed in hall measurement study as described in Sect. Topological studies (AFM).

The increase in the Eg value in (CdS + Ag + MPA) was relative to the MPA addition that was attributed to the Moss-Burstein effect^36^, which was noted for the Cu concentrations higher than 2% w/w^37^, wherein the Cu ions were located between the ZnO planes, thereby increasing the hopping nature of the charge carriers. Furthermore, even the increased conductivity in the doped films led to a bandgap reduction effect. Hence, a decrease was seen in the particulate size with the bandgap higher than (CdS + Ag) but lesser than (CdS + MPA), after MPA addition during doping.

**Figure 3:**
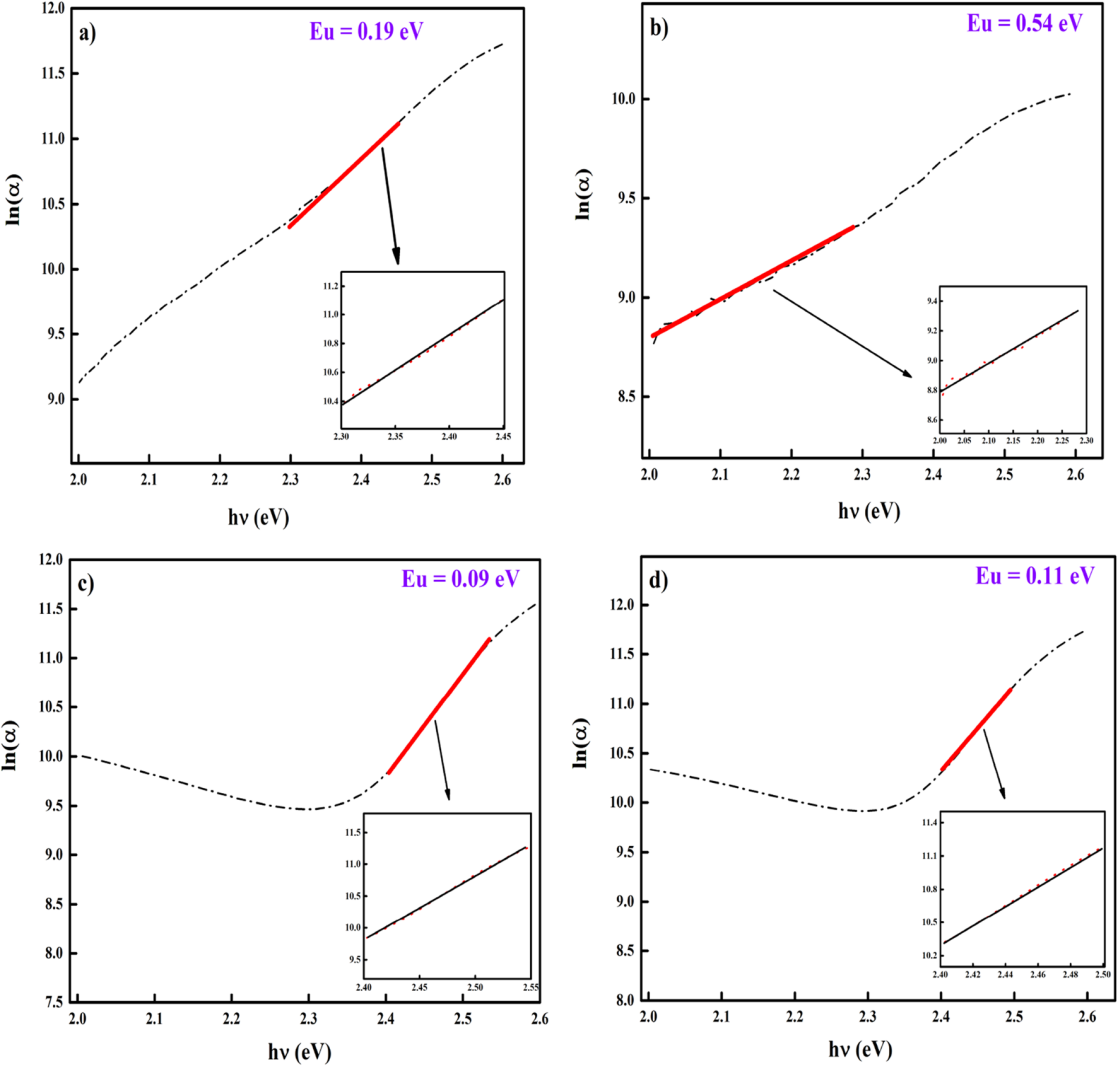
Urbach energy determined for the CdS thin films; (**a**) Basic CdS, (**b**) CdS + Ag, (**c**) CdS + MPA, (**d**) CdS + Ag + MPA.

When the photon energies were low, the spectral dependence of absorption edges followed the Urbach empirical rule^38^. The band tail width significantly affected the optical bandgap of a material. This tail was present because of the development of the localised states in the bandgap due to the disorder seen in the material. Figure 3 presents the plot of lnα versus *h*ν.

Urbach energy was controlled by structural disorder, passivation at the film surfaces and imperfections in the stoichiometry^39^. Any increase in the Urbach energy indicated an increased disorder in the structural film, which was attributed to the surface roughness resulting from doping. Furthermore, the effect of MPA appeared again, which corresponded to the lowest E_u_ value (0.09) eV, which highlighted the lower defects in the CdS structures (Table 1).

**Table 1:** Bandgap versus Urbach energy.

Samples	Band gap (eV)	Urbach energy (eV)
CdS	2.43	0.19
CdS + Ag	2.26	0.54
CdS + MPA	2.52	0.09
CdS + Ag + MPA	2.48	0.11

In the case of the deposited films, an inverse relationship was noted between Eg and E_u_, such that when E_u_ value increased, Eg decreased. This supported the explanation presented above that any increase in the bandgap energy led to a higher absorbance intensity. In other words, a higher intensity resulted in the formation of films with a higher order and small density of the localised states^40^. This relationship noted between the width of the Urbach tail and bandgap energy was similar to the relationship described by Melsheimer and Ziegler^41^ for SnO thin films, wherein they showed a decreasing band tail when the material structure shifted from amorphous to polycrystalline.

### X-ray diffraction analysis (XRD)

**Figure 4:**
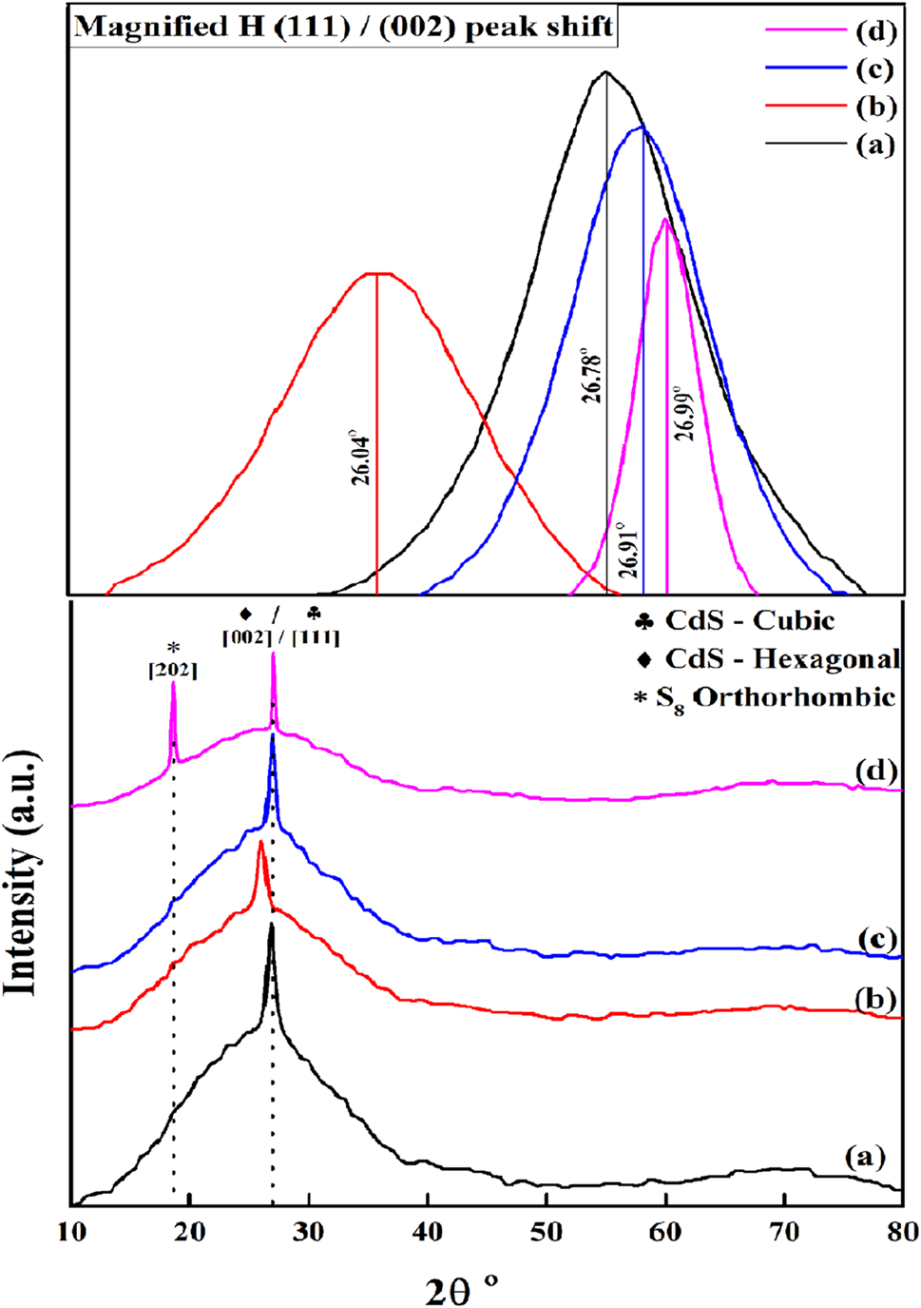
X-ray diffraction patterns of: (**a**) CdS; (**b**) CdS + Ag; (**c**) CdS + MPA; (**d**) CdS + Ag + MPA.

XRD patterns were analysed for the crystal structure of all the synthesised films^42^. These films were scanned between 10° and 80°. We observed the different phases of CdS, as XRD characteristics were described in Fig. 4.

The hexagonal and the cubic phases were the major types of CdS phases that determined the electrical properties of this material. Thus, we concluded that the dominance of one of these phases (either cubic or hexagonal) over the other phase led to the distinctive electrical properties of the material^43^.

Every sample described in Fig. 4 showed differing behaviour with regards to the mechanism of the additive that was added to the sample. The polycrystalline cubic and hexagonal forms of the CdS presented a random orientation after deposition and the samples showed numerous diffraction peaks at the relative values and intensities as described in Table 2. This can be matching also by confirming with the lattice interplanar spacing values, as we it is showed of 0.33 nm and 0.34 nm, that could be indexed to the (002) and (111) planes of both cubic and hexagonal CdS.

**Table 2:** Structural parameters of CdS thin films.

Sample	Angle 2θ degree	Intensity (a.u.)	hkl plane	The inter-planer spacing ‘d’ (nm)	FWHM ‘β’ peak width (°)	Crystallite size (nm) ‘D’	Lattice strain [%]
CdS	26.78°	4.73	(111)/(002)	0.332	0.620	13.3	1.136
CdS + Ag	26.04°	3.30	(111)/(002)	0.342	0.832	9.9	1.57
CdS + MPA	26.91°	4.84	(111)/(002)	0.331	0.576	14.4	1.051
CdS + Ag + MPA	26.99°	3.38	(111)/(002)	0.330	0.236	35.8	0.429

In the case of CdS films (Fig. 4a), a strong peak was noted at 26.78°, which was indexed to (002) and (111). This was similar to the hexagonal phase of the CdS sample (JCPDS-01-080-0006) and the cubic phase of the CdS sample (JCPDS-89-0440), respectively.

The results further indicated that the relative intensities of the CdS peak, i.e., (111) was similar in the case of the CdS and CdS + Ag thin films, except a slight shift was noted in (111) /(002) peak for the CdS + Ag films in comparison to their earlier position before doping at 2θ = 26.04°, that was oriented along (111), (JCPDS-03-065-2887). As no peak was noted for a pure silver metal, the probability of the Ag doping was due to the interstitial sites for the principal components in the structure of the CdS layer. The FWHM, which corresponds to the intense peak (111)/(002) increased with the Ag doping (shown in Table 2), presented the microstructure. It was also noted that the lattice and crystal parameters were affected by the Ag doping. This type of doping was favourable when the ionic radii of the 2 elements were comparable. The results indicated that the ionic radius of Ag^2+^ (93 Å) was comparable to that of Cd^2+^ (0.97 Å), however, it differed significantly to that of S^2−^ (1.84 Å)^44^. Hence, it was noted that the electron transfer in the lattice structure was from Ag^2+^ to Cd^2+^, which decreased the lattice constant and crystalline plane distance.

The XRD peaks of the CdS + MPA films were broad, which indicated the nano dimensions of this sample. This further showed that the particle sizes of the CdS crystals which were prepared using the MPA ligand decreased. The presence of the MPA ligand while preparing CdS did not alter the CdS crystal structure. The strong peak shifted their position at 2θ = 26.88°, indexed for (111) and (002), which suggested the cubic and the hexagonal forms. This was in agreement with (JCPDS-01-075-0581) and (JCPDS-01-080-0006) samples.

Figure 4d did not contain any peak for elemental silver, which indicated the absence of elemental Ag ion deposition. However, a new peak was noted at 18.58°, which oriented along (202). This suggested that the new structure was related to the S_8_ Orthorhombic (JCPDS-01-083-2285) sample. We attributed this result to the effect of combining MPA and Ag ions in the same chemical reaction. After comparing Fig. 4b,c, we determined that the CdS structure did not change if we added only MPA or Ag ions, however, combining these 2 ions led to the synthesis of different structures containing CdS with sulfur. After Ag^1+^ donated some electrons to the CdS films, it transformed from their oxidative state of Ag^1+^ to Ag^2+^, which returned to its primary oxidative state of Ag^1+^ by collecting an electron from the excess sulfur ions present in MPA. The S^2−^ anions acted as an electron donor to Ag^2+^. This electron donation led to the formation of an S neutral species, which reacted with the S^2−^ for forming the S_8_ orthorhombic structure.

We determined the mean crystalline sizes of different films using the Debye–Scherrer equation based on the strongest peak^45^. Table 2 presents the estimated crystallite sizes with the help of d-spacing. The results presented in the Table showed that the values ranged in the nanometre scale (9.9–35.8) nm, which indicated that the polycrystalline CdS films consisted of nanocrystal particles. In addition to crystallite size, strain in thin films is characterized as the the disarrangement of the lattice formed during deposition and is influenced by deposition conditions. Lower strain values thus suggest better crystallinity. The following equation is used to estimate the strain^46^:

1 $$\upvarepsilon =\frac{\beta }{4 tan\theta }$$

where ε = strain, β = full width half maximum (FWHM) of the reflection peak that has the same maximum intensity in the diffraction pattern, and θ = Bragg’s or diffraction angle of the x-rays.

### Film morphology

**Figure 5:**
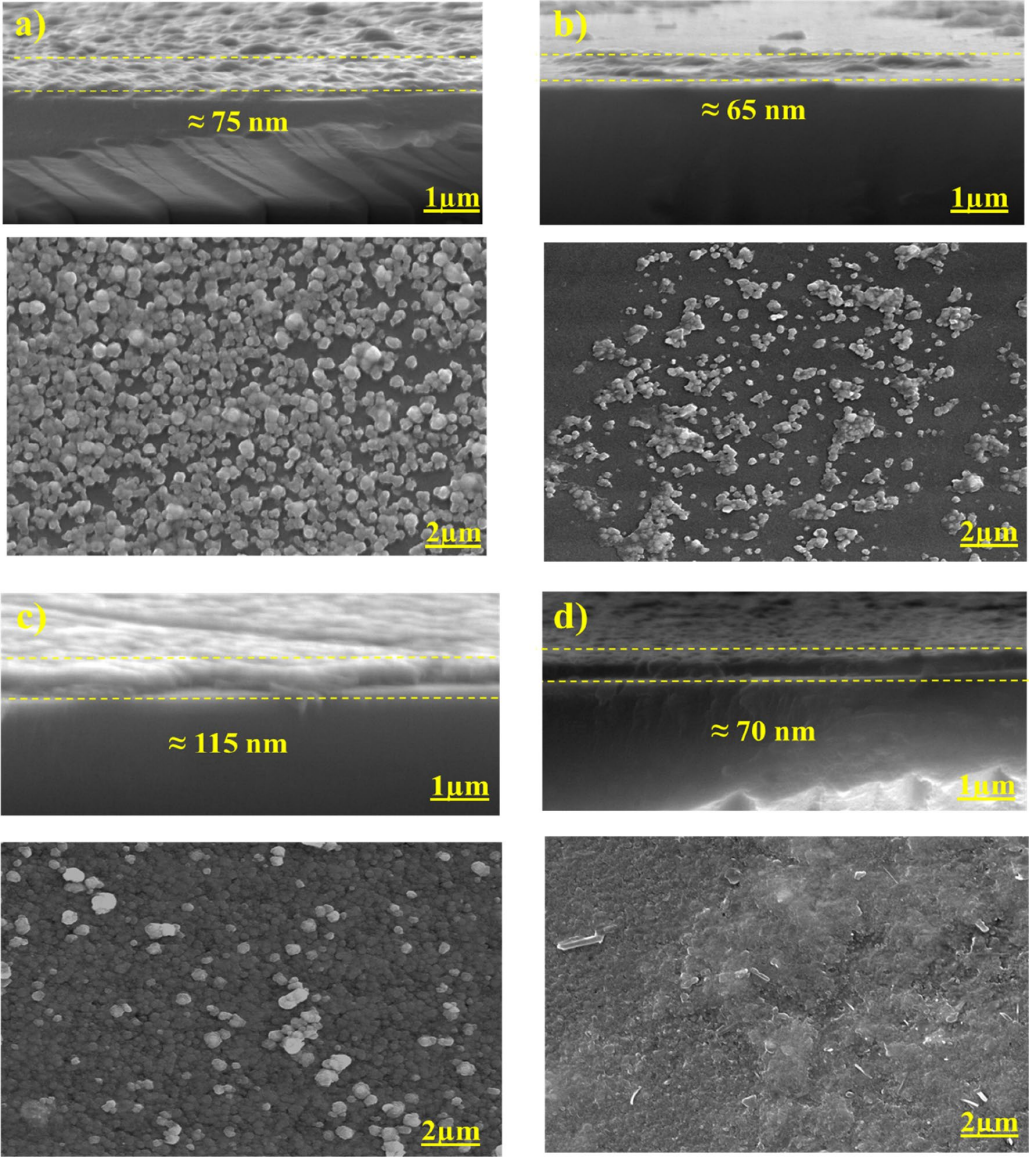
FESEM images of the top and cross-sectional view of; (**a**) CdS, (**b**) CdS + Ag, (**c**) CdS + MPA, (**d**) CdS + Ag + MPA.

It has been found that the CdS films' overall characteristics are significantly influenced by their morphology. This was particularly evident in solar cells, where grain boundaries and surface roughness had an impact on the recombination of the films. Thus, it was determined that the surface morphology and the presence of any impurities on the surface could have a impact on the operating parameters of the solar devices^47^. Figure 5 displays FESEM images of the CdS thin films.

The FESEM micrographs showed that the CdS films had a non-uniform surface and displayed less adherence onto the substrates without developing any cracks. Similar surface morphology was described in the earlier studies^48–52^.

In all cases the coverage depended on the components that are used in the reaction. In first case, CdS has been prepared without using any modification, thus the micrograph reveals that the deposited film has spheroid-like grains and the glass substrate is almost covered, with some voids here and there due to the low quality resulting from the deposition.

While in the next case, at CdS + Ag films (Fig. 5b), a non-uniform distribution of particles and non-densely packed structures is observed in the sample prepared after doping with Ag, which indicates that the component crystals grow particularly poorly and have a low crystallinity. It has been observed that, the voids between the CdS particles gets increased and uniform deposition of CdS nanoparticles is minimized. Since the film showed no uniformity, the particles formed aggregates because of these voids, which could be because of a lack of reactivity. The number of particles increases slightly without Ag doping on the CdS, and with sufficient adhesion of material particles, the surface becomes denser. Adhesion has a crucial part in the growth of thin films, which has a direct effect on power conversion efficiency. This was further ascribed to the size difference (in the ionic radii) between the Cd ions and the Ag dopant ions. The uniform distribution of Ag dopant in between the nanoparticles on the surface of CdS (for example by adding MPA) may contribute to uninterrupted passing of electron transport to the back contact of the conducting and correspondingly result in a substantially improved charge carrier density for electron conduction. Figure 5c presents the FESEM images of the CdS + MPA films. After comparing samples presented in Fig. 5a,b, it was seen that, the film was less rough with a lesser number of holes. MPA acted as a capping ligand for forming a complex, whereas the thiol-group (^−^SH) decomposed easily and acted as a sulfur source, and generating S^2−^ ions. As mentioned in the earlier studies, the formation of the intermediate metal–ligand complex inhibits the grain growth, enhances particle stabilisation and increases the optical properties of the films^53^. Furthermore, the use of ligands having terminal moieties and containing dissociable protons can lead to electrostatic separation, thereby increasing the inter-CdS distance in the films.

We also investigated its role in a larger volume of the deposited CdS films and noted that the colloids of the MPA-capped CdS were subjected to an electrostatic separation when the particles were suspended^54^. The films were seen to be thicker compared to the CdS ions deposited onto the measured region. In contrast, the thickness of the *in-situ* films that were deposited using a similar concentration of CdS was expected to become cross-linked owing to the bi-functional property of MPA. An additional increase in the sulfur concentration led to a significant change in CdS morphology. The CdS film surface changed from a granular structure to a more compact, homogenous and a dense structure with a lower particle size, which was interconnected with one another for forming a high-coverage structure. Also, the non-uniform distribution of the spherical particles was noted in Fig. 5d. The structure was altered owing to the substitution of the MPA with the Ag ions. The vacant spaces present in the Ag-doped CdS films were occupied by the MPA, which led to a very compact structure. Thus, it was noted that this combination modified the CdS film structures. The grain size was uniform and even the complete substrate was covered by the CdS film, which acted as a compact layer for preventing the leakage in the current. We explained this phenomenon with regards to an increase in the sulfur ions in the CdS host matrix, which decreased the sphere size. This is known as the growth limitation of the CdS thin films due to the saturation of the sulfur atoms in the CdS structure^50^. We determined the thickness of these films using a cross-sectional measurement and the results are shown in Fig. 5d. It was seen that the mean thickness of all the mixed films was ≈ 70 nm. When this observation was coupled with the XRD results (i.e., distinctive diffraction peaks), we hypothesised that a significant CdS deposition took place in the CdS + Ag, after assuming that the film growth occurred after the adsorption of smaller CdS particles onto the surface of the film (i.e., homogeneous growth), which was followed by excessive sulfur ions which were present in the S_8_ Orthorhombic crystal structure. XRD and the FESEM results showed that there was an increase in the size of the crystalline CdS grains when the MPA and the Ag ions were mixed, which led to a decrease in the grain boundaries and the defects. Even the crystallinity was maintained in these CdS thin films. To verify the chemical components of the film surface, we obtained the EDX spectra of each sample that had been prepared^55^, Table 3.

**Table 3:** Elemental compositions at different molar ratio (Atomic %).

Sample	Cd (at%)	S (at%)	[Cd]/[S] (at%)
CdS	59.91	40.09	1.49
CdS + Ag	60.00	40.00	1.50
CdS + MPA	60.43	39.57	1.52
CdS + Ag + MPA	62.52	37.48	1.66

The uniform distribution of sulphur ions in the sample revealed that small amounts of the sulfur-containing ligand were capped onto the surface.

### Topological studies (AFM)

**Figure 6:**
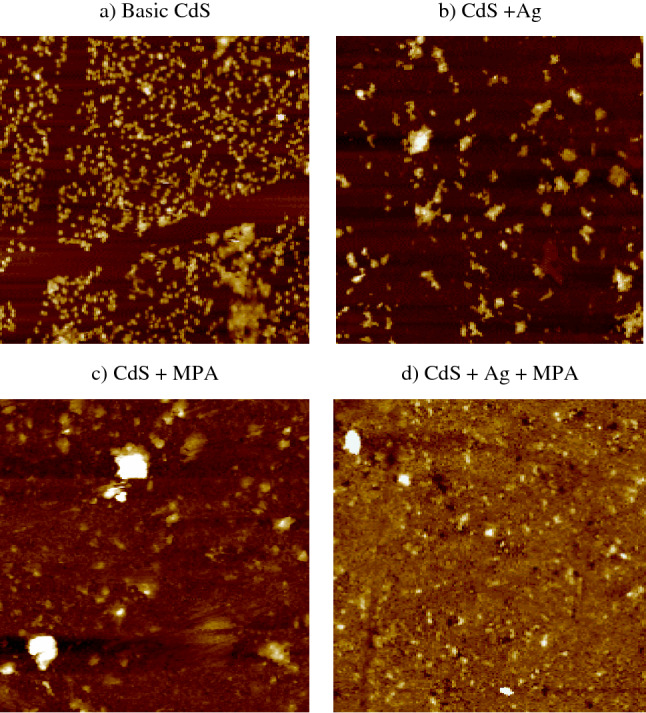
2D for thin film synthesis at: (**a**) CdS, (**b**) CdS + Ag, (**c**) CdS + MPA, (**d**) CdS + Ag + MPA.

**Figure 7:**
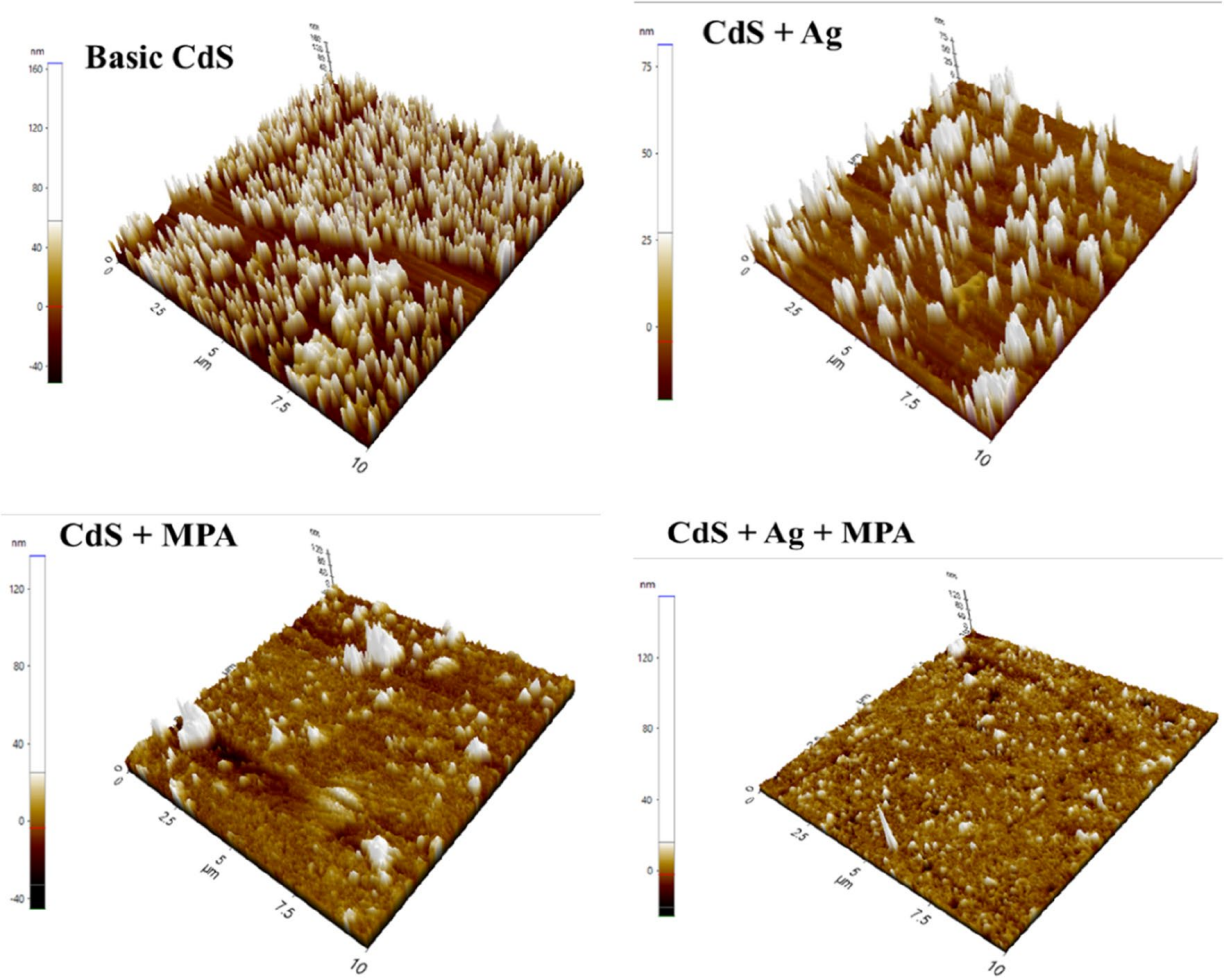
3D AFM images (10 × 10 μm) for thin film synthesis at: (**a**) CdS, (**b**) CdS + Ag, (**c**) CdS + MPA, (**d**) CdS + Ag + MPA.

The AFM method offers unique insights into CBD-grown CdS thin films' surface topological characteristic^56^. This process utilizes digital images that allow surface properties to be measured quantitatively, such as root mean square (RMS), as well as image analysis from several perspectives, including three-dimensional simulation. Analyzing the contribution of the incorporation of various materials to the film's quality is one of the purposes of AFM. AFM was utilised to display the 2-D and 3-D image topography of CdS thin film samples as an alternative growth strategy, as shown in Figs. 6 and 7. In order to quantify the overall average surface roughness, Sa, and detect particle aggregation, 10 m 10 m scan areas were used for surface topology mapping.

All surface samples have a specific grain distribution with a consistent granular topography. The surfaces are composed of nano-sized grains, and the average roughness of the deposited CdS thin films was (4–24.3) nm, indicating the formation of smooth, well-connected grains on the film (Table 4). Both FESEM and AFM images have shown the presence of small white spots throughout some clusters. It has been confirmed that white spots form, which are caused by sulphur that hasn't reacted^57^. Due to a large number of nucleation and large grain growth, there were a lot of hills, which caused the roughness to be higher^58^. The skewness value was both positive and small, near to zero.

**Table 4:** AFM analysis.

Samples	Average roughness [S_a_] (nm)	Root mean square [RMS] (nm)	Surface skewness [S_sk_]	Surface kurtosis [S_ku_]	Top ten height [S_z_] (nm)
Basic CdS (a)	24.3	29.3	1.08	3.11	215
CdS + Ag (b)	8.2	13.2	2.51	9.35	102
CdS + MPA (c)	7.4	12.9	3.64	24.71	182
CdS + Ag + MPA (e)	4	6.2	3.17	54.43	180

The findings suggest that the surface topography of sample (a) has a low smooth surface with an average roughness of 24.3 nm, in addition to a small number of summits and a low value of root mean square of 29.3 nm, due to the initial growth of CdS, which may have been caused by a low growth rate of CdS thin film or the consumption of growth solution. The characteristics of CdS fluctuate dependent on the ion concentration and the synthesis conditions, such as temperature and pH; as a result, the effects of AFM are affected by the introduction of new reaction material. All samples obtained by combining with different additives had a minimal impact on RMS.

Sample (b) indicates decreasing in the un-homogeneity of the surface with a severe decrease in average roughness value, followed by a decrease in root mean square value, due to the summit height is reduced. In addition, several dark spots are found on the CdS thin film surface as seen by the appearance of pinholes. Although the dark areas derive from the substrate, the light spots reflect the deposited particles.

It can be shown that with MPA, very thin and uniform deposition is possible. CdS deposition in MPA results in surface modification (c). The surface homogeneity of the layers is practically filled by evenly spaced grains of equal size, and the grains on the film surface seem more homogeneous and have a dense structure. There are several clusters, though, but most seem to form a close monolayer coverage. Therefore, the total reduction in roughness is 7.4 nm, which corresponds to a flat, thin-film surface.

By combining Ag and MPA samples, small particles can be observed (d). Film growth is shown to continue via the nucleation of islands that subsequently coalesce to cover the entire surface of the substrate with a dense structure. From the columnar structure of clustered particles, this can be shown. The columnar structure is a standard hexagonal CdS film structure. The findings of AFM also associated well with the results of FESEM.

### Hall effect measurement

Since CdS thin films are utilised as a buffer layer in thin solar cells, their increased conductivity may aid in efficiently separating the generated charge carriers during photovoltaic energy conversion. This consequently improves the efficiency of solar cells. To examine the maximum changes in the material's characteristics, we chose annealing temperatures between 150 and 450 °C and 10 min as the annealing period.

This investigation was comparable to one done by Akbarnejad et al. (2017), who chose annealing temperatures ranging from (300 to 500) °C^59^. CdS-treated samples exhibited higher conductivity than CdS films that had not been treated. We confirmed the n-type conductivity of CdS thin films by measuring the negative Hall coefficient values for each sample. The electrical properties of the samples at various annealing temperatures are shown in Table 5.

**Table 5:** The electrical properties of CdS thin films.

Samples	As deposited	150 °C	250 °C	350 °C	450 °C
**CdS**					
Bulk concentration (cm^−3^)	3.21E + 14	3.82E + 14	3.84E + 14	2.12E + 14	2.98E + 14
Carrier mobility (cm^2^ V^−1^·s^−1^)	12.86	10.08	12.71	17.42	15.10
Resistivity (Ω cm)	1513.46	1621.74	1278.90	1688.90	1386.46
**CdS + Ag**					
Bulk concentration (cm^−3^)	4.93E + 14	2.47E + 14	4.20E + 14	2.31E + 14	4.20E + 14
Carrier mobility (cm^2^ V^−1^·s^−1^)	11.62	14.45	10.42	16.53	11.70
Resistivity (Ω cm)	1088.25	1750.28	1426.58	1630.24	1268.91
**CdS + MPA**					
Bulk concentration (cm^−3^)	2.93E + 14	3.65E + 18	7.88E + 17	3.53E + 14	2.00E + 15
Carrier mobility (cm^2^ V^−1^·s^−1^)	14.00	0.94	1.22	21.35	12.70
Resistivity (Ω cm)	1521.76	1.81	6.47	826.94	244.70
**CdS + Ag + MPA**					
Bulk concentration (cm^−3^)	2.14E + 17	1.87E + 17	6.90E + 17	1.93E + 14	4.28E + 14
Carrier mobility (cm^2^ V^−1^·s^−1^)	2.17	0.98	0.30	8.08	12.38
Resistivity (Ω cm)	13.36	33.77	30.04	4004.30	1178.82

The results presented in the Table showed a significant increase in the carrier concentration with a reduction in the mobility and resistivity, when the CdS films were doped, in situ, during deposition. An increase in the Cd and Ag vacancies, because of the Ag concentration, was responsible for improving the electrical properties of the material after Ag doping. The Ag vacancies associated with the Cd and S vacancies lead to formation of photosensitization centers in the CdS matrix^58^. The presence of the Cd and S vacancy sensitisation centres were ascribed to the lattice defects in the non-treated films, hence, we stated that the increased photosensitivity of the films was because of the formation of additional Ag vacancies in the above films after their doping. Typically, a process called doping is used to improve the conductivity of a material. Doping is simply the deliberate introduction of impurities into a semiconductor crystal to generate more free carriers. Incorporating impurity atoms, such as Ag, into semiconductor crystals results in the formation of an extra energy level, typically towards the edge of the conduction or valence band. Transition metals in the periodic table, such as Ag, which have one valence electron, are known as donor impurities that introduce an extra energy level towards the conduction band's bottom. This resulted in CdS + Ag, which exhibited the lowest absorption edge at around 548 nm. Since Cd can only share two electrons with the surrounding Ag atoms, an additional electron from the Ag is not shared with any atoms and tends to pass into the conduction band with low thermal energy, forming Ag^+2^. Consequently, the concentration of electrons in the conduction band is raised, and the density of electrons transferred into the conduction band is typically equal to the doping concentration, assuming complete ionisation of impurity atoms. Overall, doping increases the conductivity of semiconductors by increasing the density of free charge carriers.

When MPA was added to the samples, it increased the number of OH^−^ molecules in the reaction, which further increased the S^2−^ ion concentration. This, in turn, increased the deposition rate and enhanced the thickness of the thin films. Thus, a highly-conducting film showed a lower resistivity. A high carrier concentration was attributed to the material properties like bandgap narrowing, decreased grain boundary and dislocation density, and increasing grain sizes of all films^60^.

The increase in carrier concentration from 3.21E + 14 (in the case of pure CdS) to 2.14E + 17 (cm^−3^) after the incorporation of Ag-doped CdS indicated that the addition of Ag impurities acted as a strong donor after MPA was mixed in the sample, in comparison to the Ag ions alone. This also highlighted another effect of their combination. The CdS + Ag + MPA films also displayed a lower ρ value, 13.36 Ω cm, which was attributed to the higher incorporation of mixed additive ions into the lattice, because of the higher solubility of all doping salts into the growth solution.

In an earlier study, we carried out a few post-deposition treatments which decreased the electrical resistivity of the CdS films^15^. Some of the earlier studies have observed that the crystallinity of the structures improved with an increase in the annealing temperature, due to the microstructural defects, thereby leading to an increase in the carrier concentration^38^.

When air annealing was carried out for the CdS + Ag films, deterioration was noted in their electrical properties. The resistivity changed from 1513.46 Ω cm (for the films without any doping) to 1088.25 Ω cm for films which were doped with Ag ions. This value increased to 1750.28 Ω cm at the annealing temperature of 150 °C. This result was noted due to the presence of impurities like oxygen ions, which affected the band structure of all films, as it formed the recombination and trapping centres. This imbalance between the recombination and trapping centres was responsible for the photo response of these films after illumination.

In the case of the CdS + MPA and the CdS + Ag + MPA films, the results of different annealing temperatures (ranging between 150 and 350 °C) highlighted the improved electrical properties of the CdS films. We determined the dependence of the carrier concentration for CdS + MPA films to the level of 3 orders of magnitude, ranging from 10E + 14 to 10E + 18 cm^−1^, at 150 °C. A higher conductivity was associated with the chemisorbed oxygen ions present in the grain boundaries of all films, owing to the grain growth. When oxygen diffused in the material because of the temperature effect, it interacted with the CdS vacancies, arising because of the thermal breakdown of the CdS or lattice defects, and yielding conductive CdO^61,62^. However, when the annealing temperature was increased to ≤ 350 °C, the CdS concentration increases, which decreases the film conductivity. Furthermore, an undesirable property that was noted at the annealing temperatures higher than 450 °C was that the Cd and/or S atoms underwent the “out-diffusion” process. Here, the existing compounds can breakdown to their constituent atoms that diffuse and freely interact outside or inside the CdS matrix. Thus, all samples, except the CdS + MPA films showed a higher resistivity. The results indicated that the CdS films, grown using this technique, displayed better charge carrier transportation despite having a low crystallinity in some of the cases. Therefore, the low-resistivity of the CdS thin films allowed their application as buffer layers in the CZTS and CIGS photovoltaic devices.

## Growth mechanism

Though the CdS films are generally prepared using simple chemical bath deposition (CBD), very few researchers described the theoretical and experimental justification of the growth mechanism used for depositing the films.

The general chemical reaction is as follows:

$$\begin{gathered} {\text{CdSO}}_{4} + 2{\text{NH}}_{4} {\text{OH}} \to {\text{Cd(OH)}}_{2} + ({\text{NH}}_{4} )_{2} {\text{SO}}_{4} \quad {\text{ - - - - - - - - }}\quad {\text{Decomposition}}{\mkern 1mu} {\text{Cadmium}}{\mkern 1mu} {\text{salt}} \hfill \\ {\text{Cd(OH)}}_{2} + 4{\text{NH}}_{4} {\text{OH}} \to {\text{Cd(NH}}_{3} )_{4}^{{2 + }} + 2{\text{OH}}^{ - } + 4{\text{H}}_{2} {\text{O}}\quad {\text{ - - - - - - - - }}\quad {\text{Formation}}{\mkern 1mu} {\text{intermediate}}{\mkern 1mu} {\text{complex}} \hfill \\ ({\text{NH}}_{2} )_{2} {\text{CS}} + 2{\text{OH}}^{ - } \to {\text{CH}}_{2} {\text{N}}_{2} + 2{\text{H}}_{2} {\text{O}} + {\text{S}}^{{2 - }} \quad {\text{ - - - - - - - - }}\quad {\text{Decomposition}}{\mkern 1mu} {\text{thiourea}} \hfill \\ {\text{Cd(NH}}_{3} )_{4}^{{2 + }} + {\text{S}}^{{2 - }} \to {\text{CdS}} + 4{\text{NH}}_{3} \quad {\text{ - - - - - - - - }}\quad {\text{Formation}}{\mkern 1mu} {\text{CdS}} \hfill \\ \end{gathered}$$

The overall reaction:

$${\text{CdSO}}_{4} + 6{\text{NH}}_{4} {\text{OH}} + ({\text{NH}}_{2} {)}_{2} {\text{CS}} \to {\text{CdS}} + {\text{CH}}_{2} {\text{N}}_{2} + 4{\text{NH}}_{3} + 6{\text{H}}_{2} {\text{O}}$$

**Figure 8:**
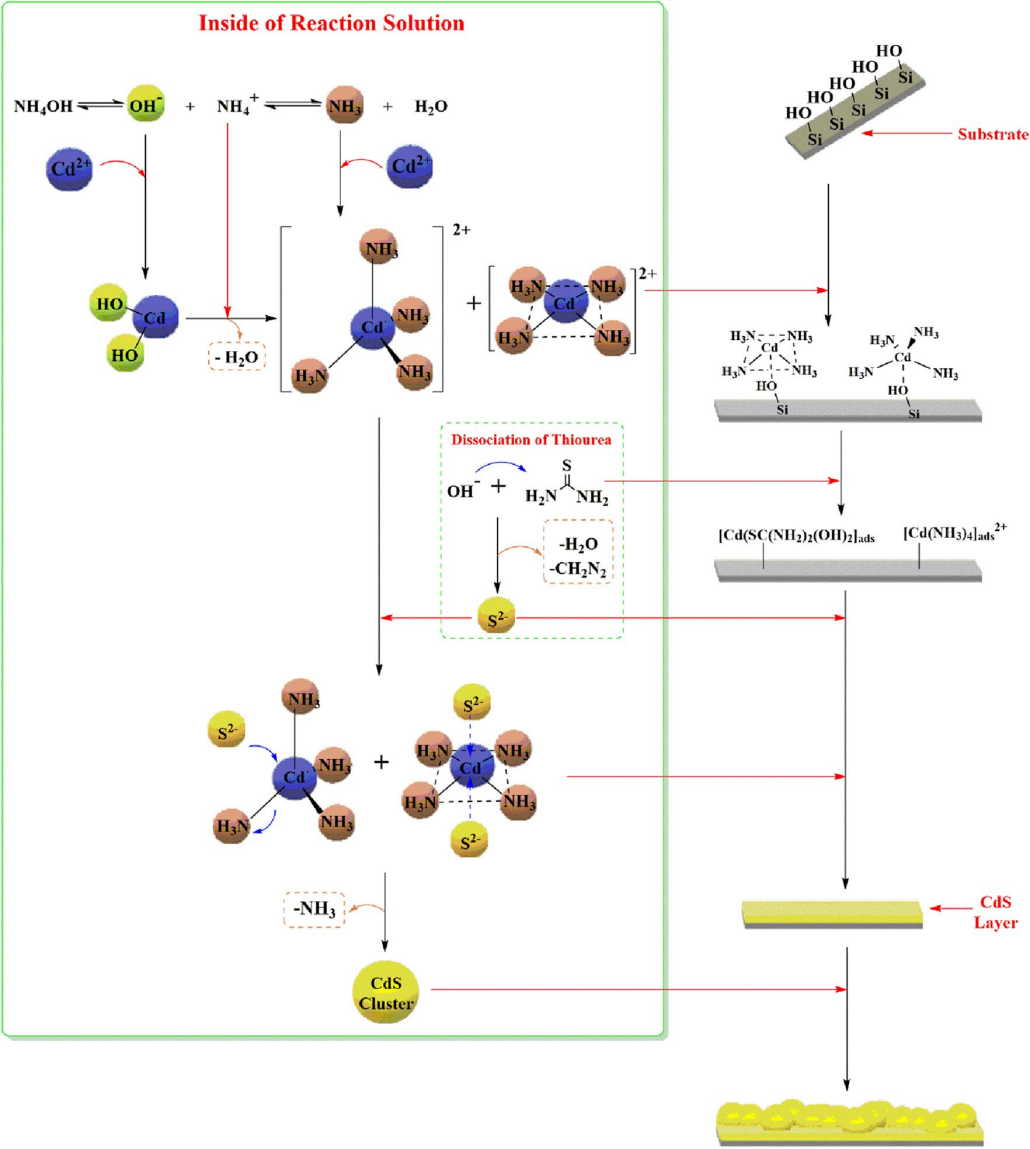
The growth mechanism of basic CdS in CBD.

The following scheme presents the primary mechanism of the complete CBD deposition technique in Fig. 8.

In CBD process, the chemical reaction is conducted in an alkaline solution to prevent the precipitation of the metal hydroxide ions as well as to decrease the concentration of the free metal ions in the reaction bath. This prevents the accumulation of the desired product^63^. One of the most important factors influencing CdS preparation and precipitation is pH. In this study, we selected ammonium hydroxide as the coordination agent. The CdS preparation reaction system's basic media is critical for the formation of intermediate complexes required for the formation of the CdS film. As decrease in the pH of the reaction system causes an increase in the concentration of free cadmium ions within the solution^64^. The pH ranges most suitable for preparing these CdS films was (9–11) to 11, while 10 is the optimal pH which giving a majority for the cubic geometry of the crystal structure^65^. An increase in pH above 12 reduces the crystallinity of the CdS film and results in an amorphous CdS film^66^. Cadmium intermediates formed prior to combining with sulfur ions are critical in preparing a homogeneous CdS film with higher crystallinity. Cd(OH)_2_, (square planar and tetrahedral) of [Cd(NH_3_)]_4_^2+^, [Cd(OH)_2_SC(NH_2_)_2_]_ads_ and [(NH_3_)_3_Cd–]^2+^–OH-Site are an important intermediate compound formed during the preparation of CdS film when using NH_4_OH for setting the pH system of the reaction.

Hence, when the pH of the reaction bath is gradually increased, the metal complex becomes more stable as it decreases the availability of the free metal ions in the solution. Furthermore, the alkaline environment which is created by the buffered solution enables (NH_2_)_2_CS to release S_2_^2−^ ions and allows the presence of Cd^2+^ as [Cd(NH_3_)_4_]^2+^, which ensures that the complete reaction takes place slowly and uniformly^67^. With regards to the bath temperatures, the optimal temperature ranges between 70 and 80 °C, and it can improve the photovoltaic quality of the CdS thin films^68^. At this temperature, the complex dissociates very efficiently in the reaction bath. This further increases the kinetic energy of all molecules, which increases the interaction between the ions. This can increase / decrease the terminal thickness of the films, based on the level of supersaturation of the reaction solution^69^. Based on the modern complexity theory, when ammonium is used in the reaction, the common Cd^2+^ complex structural geometries noted in the crystals are the tetrahedral, square planar, and octahedral geometries^70^. However, the tetrahedral and the square planar geometries of the Cd^2+^ complex with NH_3_ was more stable compared to the octahedral geometry because the thermodynamic stability of the complex having an octahedral structure was lower compared to the tetrahedral and square-planar complexes^71^. The stability of the tetrahedral and square-planar complex can to some extent prevent the crystallites from showing an epitaxial growth, which further increases the homogeneity of the CdS films^72^. Another advantage that was noted for the square-planar and tetrahedral complex geometries was that it decreased the steric and electrostatic hindrance factors based on the order of substitution of the S^2−^ and NH_3_ ions which took place at the below and top faces of the complexes. Furthermore, ammonium hydroxide is seen to be a strong base which prevents the coordination of the nitrogen atoms in thiourea with the Cd^2+^ ions and ensures the balance with the Cd(OH)_2_. Also, the presence of a Cd(OH)_2_ complex provides sufficient time for forming a homogenous film wherein the 2 complexes, i.e., [Cd(OH)_2_ $\leftrightarrow$ Cd(NH_3_)_4_^2+^] are seen to be in equilibrium. Based on Le Chatelier's principle, this equilibrium is more inclined towards a stable complex present in the chemical solution (from Cd(OH)_2_ to Cd(NH_3_)_4_^2+^).

Cadmium orbital ions Cd(II) have empty 5 s and 5p positions that are not occupied by electrons. As a result, cadmium ions react to form tetrahedral complexes with sp^3^ hybridization, resulting in the formation of a hexagonal CdS crystal structure. Due to the solution that comes into close contact with the glass wall, gains additional thermal energy, an electron pair is transferred to the empty 5 s orbit from a dx^2^–y^2^ orbital to form the square planar intermediate complex. This displacement enabled the orbital dx^2^–y^2^ to converge energetically from the three 5p orbitals and combined with the orbital to generate orbits having 2D dimensions on the X-axis and Y-axis. The combination of the orbitals (dx^2^–y^2^–5p) decreases the electric repulsion of all close orbits filled with the electrons (4d orbital). Thus, the substitution reaction takes place when the negative sulfur (S^2−^) ions attack the positively-charged ionic nucleus of the Cd^2+^ complexes [Cd(NH_3_)_4_]^2+^ and the ammonia ligands are replaced by the S^2−^ ions.

The XRD results indicated the presence of many configurations in the CdS layers arising due to the random arrangement of intermediate complex molecules on the slide surface. This leads to the attack of the sulfur ions on the [Cd(NH_3_)_4_]^2+^ tetrahedral and square planar complexes and leads to the development of the CdS layers. This process causes precipitation of the unreacted sulfur ions between the CdS layers or their surface, along with the fact that the CdS tends to form a cubic structure rather than a hexagonal structure. The ionic and the colloidal mechanisms show homogenous nucleation. It was noted that the film-forming mechanism was more complex compared to the primary mechanism of the complete cluster. The general mechanism, i.e., both the basic and film-forming mechanisms, are based on the assumption that the sulfur ions are adsorbed onto the surface of a solid Cd(NH_3_)_4_^2+^ complex, for forming a metastable surface complex compound, which gets decomposed after the formation of the CdS.

Initially, the tetrahedral and the square planar complexes of the Cd(NH_3_)_4_^2+^ were adsorbed onto the SiO_2_ substrate surface by an oxygen-containing substrate, which yields a common layer containing Cd(NH_3_)_4_^2+^/SiO_2_. The variation in the polarity between the oxygen atoms present in this substrate and complexes allow the adhesion of these complexes and their formation by the initial layers. However, in the case of the square planar complexes, the interaction with the SiO_2_ is direct and occurs from any direction of this complex. With regards to the tetrahedral complexes, the interaction eliminates one ligand molecule in every tetrahedral complex owing to the steric hindrance effect. Stage 2 includes the introduction of a sulfur source and release of sulfur ions in the reaction solution. These ions come into direct contact with the atomic layer formed onto the substrate’s surface. This interaction causes the formation of a precursor or intermediate layer, i.e., CdS/Cd (NH_3_)^2+^/SiO_2_.

**Figure 9:**
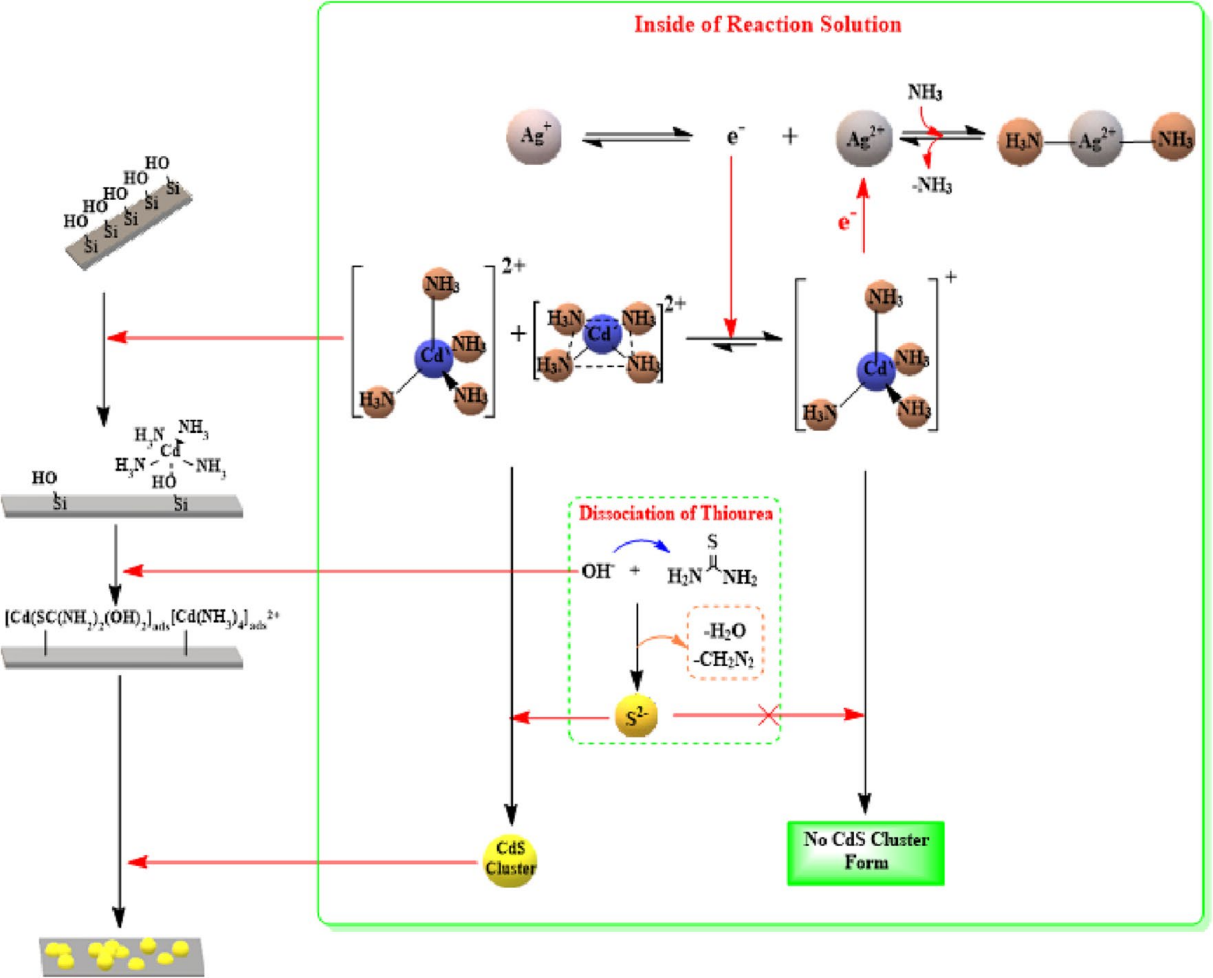
The growth mechanism of CdS + Ag in CBD.

Many researchers have expressed their desire to use doping in-situ processes since these techniques can theoretically lead to the development of marginal broad absorption bands which are closer to the band edge. Introducing Ag ions into the film can help in creating n-type materials wherein a majority of the carriers were electrons. A comparison of the basic mechanism for the CdS film formation indicated that the Ag ions can form a square planar geometry, which is initially unstable in a solution owing to the formation of the symmetrical bonds. However, 2 short symmetrical bonds can become stable after the introduction of the silver ions^73^. Generally, it was noted that the silver ions favoured a linear structural geometry because of the lack of a stereochemical directionality which is formed from the d^10^ configuration and a relatively weak nature of the silver(I)-ligand bonds^74^. Hence, the most stable geometrical structure was seen to have a linear geometry^75^ as the ground and the excited state energies for this geometrical structure were very stable^76^. In this case, one Ag ion was donated to the Cd^2+^ ions. This charge transition from the Ag^+^ ions to the Cd complexes altered the structure of the [Cd(NH_3_)_4_]^2+^ complexes (having a square planar geometry) to the [Cd(NH_3_)_4_]^+^ complex (which formed the tetrahedral shape) and led to the formation of unstable [Ag(NH_3_)_2_]^2+^ linear complexes, Fig. 9.

The electron transition between the shape and charge can alter the core structure of the Cadmium ions. The electrons that are transferred to the 4d (to the orbital with lower energy 4dx^2^–y^2^) or 5 s, helped in reordering the 4NH_3_ ligands and coordinating with the new orbital, i.e., 6 s. This different arrangement helped in the formation of the sp^3^ hybridisation and yielded molecules with a tetrahedral shape, which was the dominating shape in the solution. The linear shape of the [Ag(NH_3_)_2_]^+^ molecules enabled their interaction with the [Cd(NH_3_)_4_]^2+^ complexes and the electron transfer with the help of the ammonia ligands. In the case of the cadmium cations, a higher oxidation state led to the formation of stable complexes in comparison to the low oxidation states with the NH_3_ ligands^77^. Hence, electrons are lost rapidly from the [Cd(NH_3_)_4_]^+^ complexes and are transferred to the Ag^2+^ (which is unstable in the 2 + oxidation state and returns to the 1 + state). This is further transferred to the new molecule, i.e., [Cd(NH_3_)_4_]^2+^, which displays a similar resonance state between the molecules. Such transitions prevent the formation of the S^2−^ and also block the reaction of the Cadmium cations with S^2−^. The XRD results showed that the (CdS + Ag) films display a hexagonal structure, formed majorly of tetrahedral complexes, which allow the film to display a hexagonal structure.

**Figure 10:**
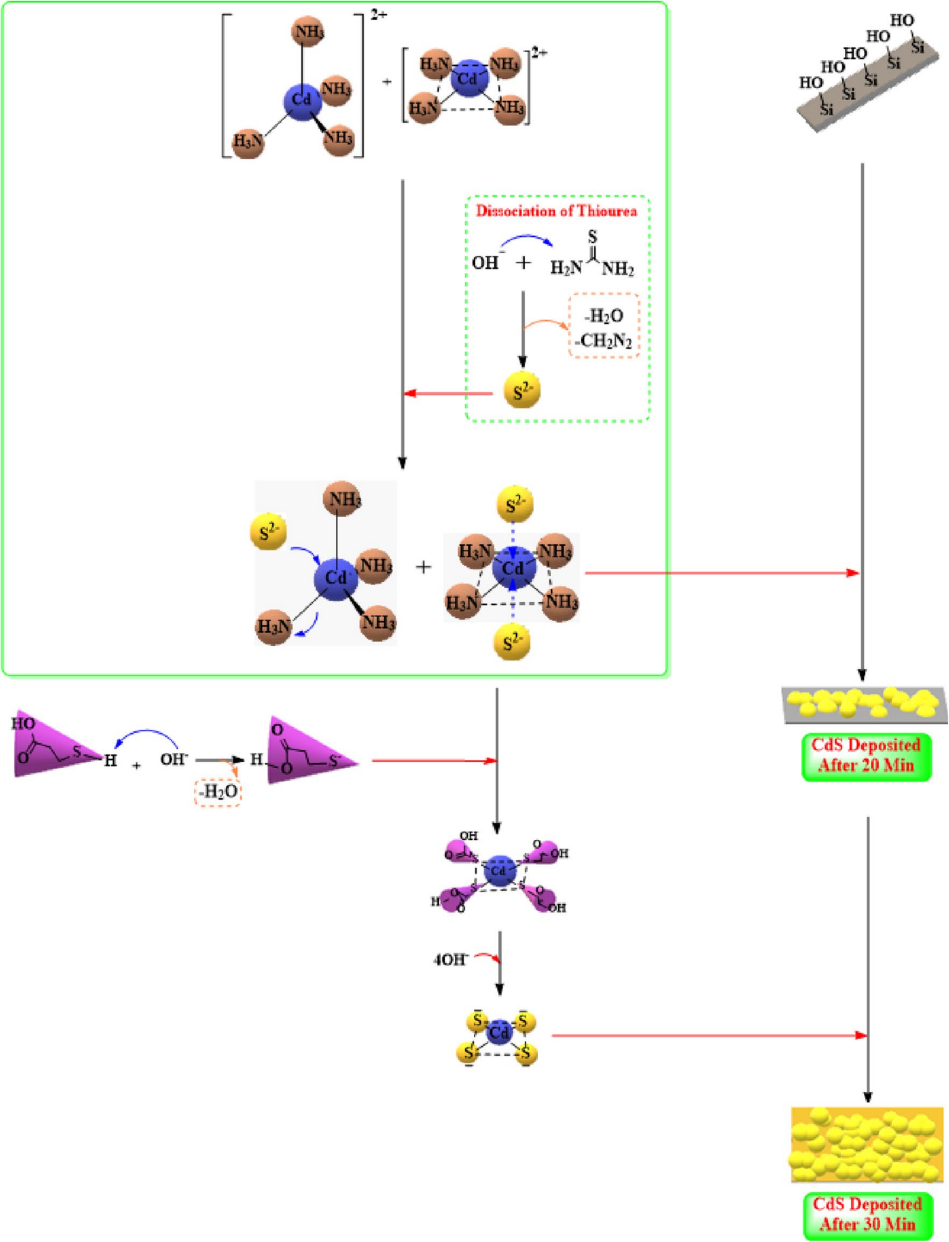
The growth mechanism of CdS + MPA in CBD.

As mentioned above, the CdS thin films can directly be formed onto the substrates, however, if the complex cations were insufficient, the colloidal Cd(OH)_2_ is generated which reacts with the S^2−^ ions for decreasing the concentration of free Cd^2+^ ions. Here, the addition of more ammonia helps in controlling the OH^−^ ion concentration in the solution and forming a tetra-amino-cadmium complex. This methodology can yield poor quality CdS films which cannot adhere properly to the substrates. Hence, in this study, we used organic substrates for preventing the aggregation of the particles after capping their surfaces. MPA used as the stabiliser since it is a strong chelating agent and contains a thiol group (^−^SH) (which is a simple hydrophilic group) that can bond the nanoparticles and organic molecules by forming covalent bonds between the free electrons in the S atoms of the thiol group and empty orbitals of the surface Cd atoms present in the CdS nanoparticles^78^. The coordination of -SH groups with Cd^2+^ ions has a significant advantage as it prevents CdS agglomeration and aids in the reduction of dangling bonds in the surface^79,80^ as shown in Fig. 10.

MPA reacted with the intermediate square planar complexes which were present in the solution. This square planar shape of the complexes allowed the replacement of the ammonia ions by the sulfur ions. This attack took place from top to bottom of this plane, while the ammonia ions are left alone. Owing to the electron density of MPA, it cannot interact with the intermediary tetrahedral complexes; however, if it interferes with the tetrahedral complexes, it will interact with the intermediary tetrahedral complexes present in the solution and not the surface. This will induce the Cd ions in the centre for forming a square planar complex before depositing it onto the surface. Based on the Hard or Soft Acid/ Base (HSAB) theory, the Cd^2+^ ions are seen to act as a soft acid, while the MPA possesses bi-functional linkers, i.e., -S- and -COO- groups^81^. As a result of the possibility of polarization of the thiol group's electronic cloud by the cadmium metal, the thiol group in the MPA compound became the major bonding group that binds to the metal, and the carboxylic group could not be alone with the centre metal cation^82^. Hence, the Cd^2+^ ions can coordinate better with a soft base thiol group which is present in MPA. The ammonia ligands are considered as the mono-dentate coordination agent ligands, wherein the mono-dentate ligands form a complex with the metal ions which are less stable than those formed between the metal ions and bidentate and tridentate ligands. Thus, the intermediate complex, i.e., [Cd(NH_3_)_4_]^2+^ assisted in the reaction rate and increased the precipitation of the CdS ions. In this case, the particle growth can be regarded as the polymerisation process, wherein the capping molecules can act as the chain terminator. Furthermore, when the precipitation is incomplete, owing to the strong adhesion between the Cd^2+^ ions and (^–^S) groups, it can result in the formation of small-sized particles. Hence, the capping agents are seen to form a stable and strong bond with the nanoparticle surfaces, which helps them remain permanently attached to the particles without interfering in the processes related to the nanoparticles^83^. In this study, the XRD results indicated that the MPA-capped CdS nanoparticles showed the smallest particle size, which highlighted the fact that the chemical interaction between the thiols and CdS nanoparticles was one of the strongest that was noted amongst all the studied mechanisms. The complexation factor plays an important role in the formation of CdS surface morphology and its deposition on the substrate^84^. Thus, the addition of MPA improved the reaction rate and offered a sufficient concentration of sulfur ions for increasing their probability of colliding with the Cd^2+^, thereby yielding a homogenous CdS thin layer surface and uniform pore diameter, after their reaction with the ends of thiol groups^85^.

**Figure 11:**
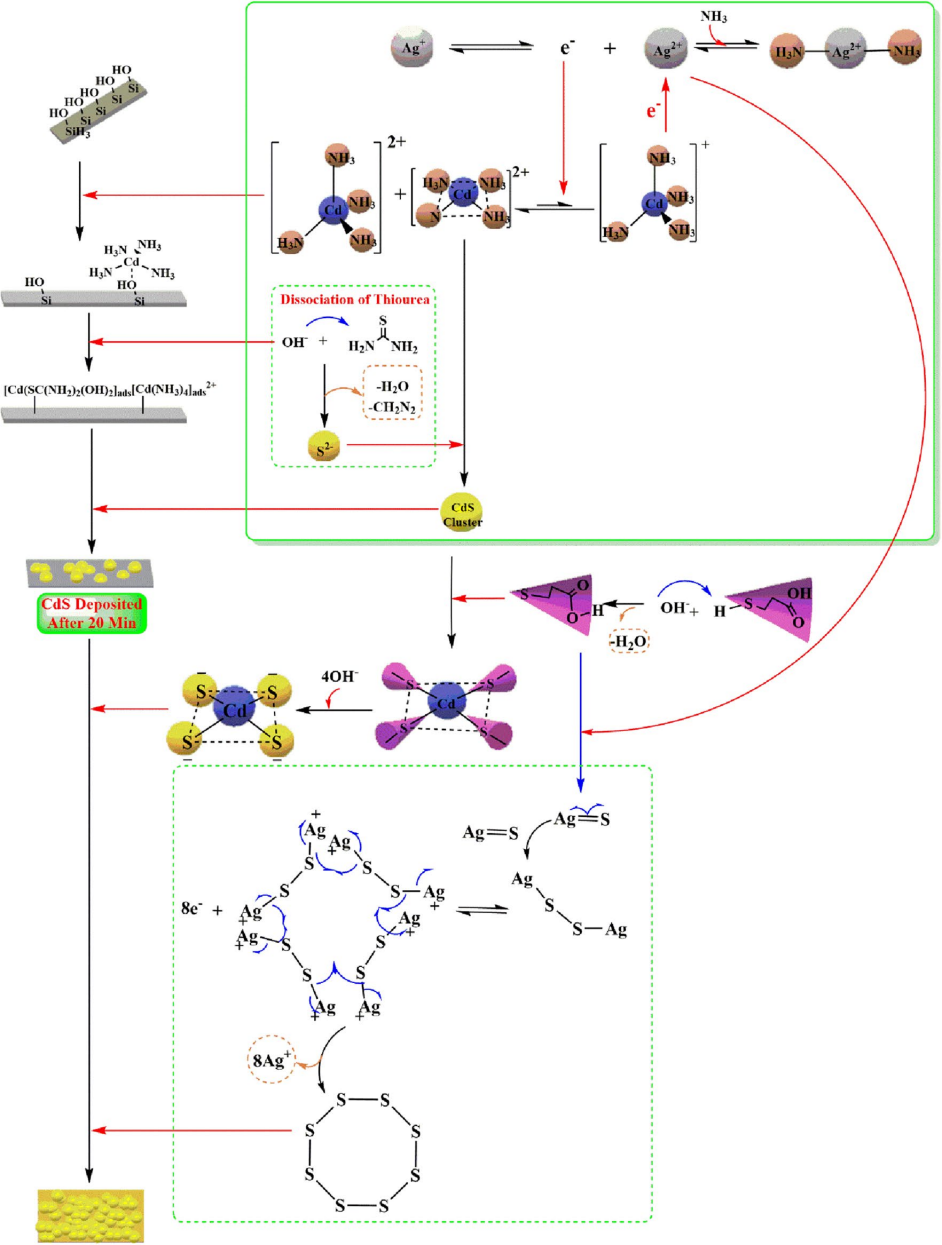
The growth mechanism of CdS + Ag + MPA in CBD.

Preparation of small particles with systematically controlled size yields a compact and homogeneous layer due to the surface complexation reaction was seen to be an appealing technique. Hence, in this study, we developed a new systematic process which combined every mechanism. We determined how the various mechanisms were related to one another. During the Ag^+^ doping process, the formation of the CdS thin films was affected by the Ag^+^ ions owing to the transition of the electrons from the Ag^+^ ions to the Cd^2+^ ions, and vice versa. In the case of the MPA, only the square planar complexes could be induced. The combination of these approaches led to the formation of a homogenous CdS surface. Along with offering a secondary sulfur ion source, the MPA inhibited the effect of the Ag^+^ ions in the chemical reaction, which helped in the formation of the orthorhombic structures^86^. In S_8_, a crown-like structure was noted which involved all the ‘s’ atoms in the single disulphide bond formation between one another using the 3p_y_, 3p_z_ electrons and attaining stability^87^. With regards to the equilibrium between the Ag^+^ and Cd^2+^, the Ag^2+^ ions coordinated with the thiol (HOOCCH_2_CH_2_S^−^) group present in MPA after donated protons to the NH_3_ base. The (HOOCCH_2_CH_2_S)_2_Ag coordination is destroyed since the Ag^2+^ complexes were unstable and the MPA ligand could be readily disassociated and released radical mono-anions S_n_·- into the solution^88^. These radical mono-anions (S_n_·^−^) can combine with other radical mono-anions S_n_·- for forming the crystalline S_8_ Orthorhombic structure. It was also noted that the MPA (HOOCCH_2_CH_2_S^−^) could react with the Cd^2+^ ion residues. The resistance which was displayed by the MPA to a silver effect was noted on the XRD spectra. A sharp XRD pattern showed that the CdS composition was the mixture of the hexagonal and cubic structures, where an increase in the crystallinity was noted due to the formation of the S_8_ in the CdS composition, Fig. 11.

## Conclusions

CdS films' electrical and structural characteristics were strongly influenced by the growth mechanism and growth parameters. We developed a linker-assisted chemical bath deposition process for synthesising CdS films. We studied the spectral and physical properties of the CdS films developed using different synthesis techniques, which could predict the CdS thin layer chemical synthesis mechanisms. The optical analysis reveals a variable bandgap between 2.26 and 2.52 eV. The XRD investigation revealed that the CdS thin films crystallised in two main structural phases, namely cubic and hexagonal wurtzite structures, with a preferred orientation along the (111/002) reflection plane. In addition, the morphological analysis of these films revealed that the CdS + MPA sample had spherical grains that were uniformly distributed. The results indicated that the major influence of this technique in the electrical properties of CdS films occurred after annealing, particularly at 150 °C, since the CdS + MPA showed a jump of 3 orders of magnitude to 3.65E + 18 cm^−1^, and 1.81 cm^2^ V^−1^·s^−1^ for the carrier concentration and resistivity, respectively. Additionally, the inclusion of MPA in this reaction, as a source of sulfur ions and linker agent, helped in regulating the CdS formation. In the presence of MPA and silver ions, the MPA was a reducing agent and affected the silver-based reactions as led to some side reactions which yielded S_8_.

After analysing the different films associated with the CdS-CBD modified approach, we determined that the combined CdS films have a crystalline structure with higher carrier concentrations.Therefore, the approach proposed in this work was preferred for achieving a more efficient charge carrier transport.

## Data Availability

The datasets used and/or analysed during the current study available from the corresponding author on reasonable request.
